# Supplementary material for: Application of sodium-ion-based solid electrolyte in electrostatic tuning of carrier density in graphene
Source: Sci Rep. 2017 Jun 9;7:3168. doi: 10.1038/s41598-017-03413-5 (PMC5466694; doi:10.1038/s41598-017-03413-5)
Supplement: Supplementary file 1 — supplementary material [file 41598_2017_3413_MOESM1_ESM.pdf]

Supplementary Materials for

Application of sodium-ion-based solid electrolyte in electrostatic tuning of carrier density in graphene

Jialin Zhao[1,2,3*], Meng Wang[1,2,3*], Xuefu Zhang[1,2], Yue Lv[1,2,3], Tianru Wu[1,2], Shan Qiao[1,2,3], Shufeng Song[4†], Bo Gao[1,2,3†]

[1]State Key Laboratory of Functional Materials for Informatics, Shanghai Institute of Microsystem and Information Technology, Chinese Academy of Sciences, 865 Changning Road, Shanghai 200050, China.

[2]CAS Center for Excellence in Superconducting Electronics (CENSE), Shanghai 200050, China

[3]University of Chinese Academy of Sciences, Beijing 100049, China

[4]College of Aerospace Engineering, Chongqing University, Chongqing 400044, P.R. China

E-mail: sfsong@cqu.edu.cn and bo_f_gao@mail.sim.ac.cn

This file includes:

Fig.S1 and Fig.S2

We also tried to fine-polish Na^+-SE substrates using oil-based lubricants. The surface roughness was reduced to an approximate value of 20 nm as shown in Fig. S1a. The fine-polished substrates usually show similar carrier density tuning capability as those hand-polished substrates. Fig.S1b demonstrates the carrier density variation of a single-layer graphene sample transferred onto a fine-polished substrate. The change of the electron density is also in the order of 10^{14} cm^{-2}. However, we also found that some of the single-layer graphene samples transferred onto the fine-polished substrates showed irregular electronic transport behaviors. Fig.S2 shows the Resistance vs. Gate voltage curve of one such sample. The double-resistance-peak structure is likely due to the inhomogeneous surface chemical doping of the substrate to the graphene sheet. We are trying to find better lubricants to improve the polishing process.

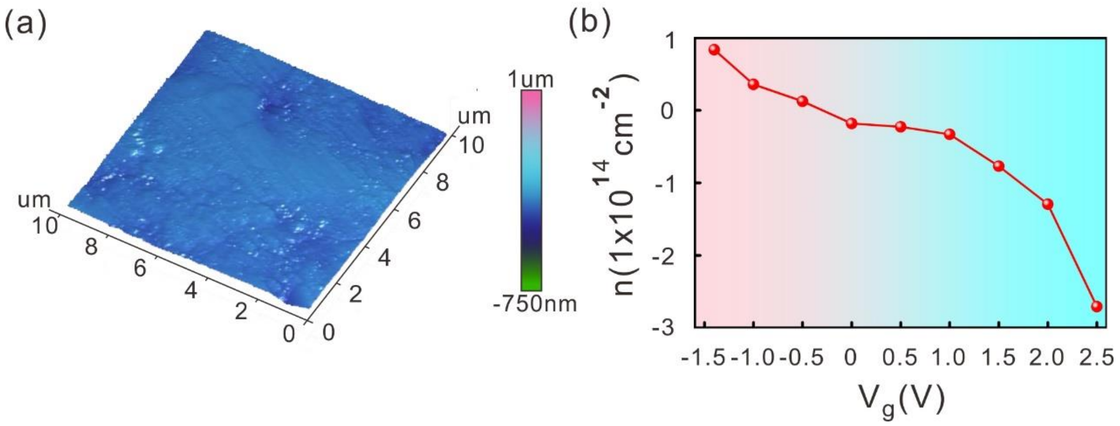

Fig.S1: (a) Atomic Force Microscope image of a fine-polished Nasicon-type solid electrolyte substrate. (b) The carrier density variation

of a single-layer graphene sheet as a function of the back gate voltage. The measurements were performed at room temperature.

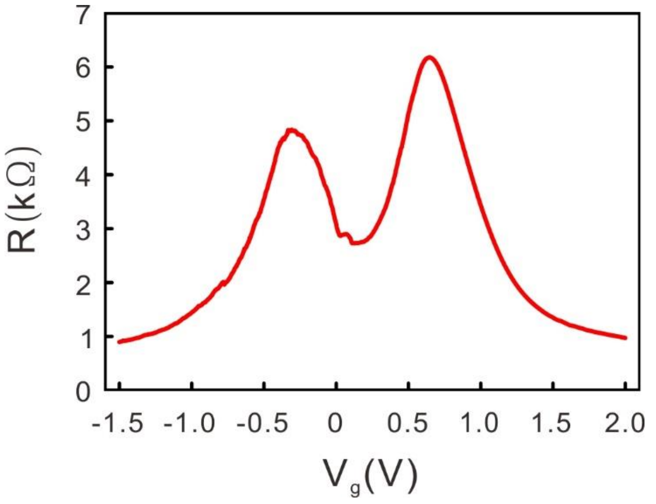

Fig.S2: The irregular resistance variation with the back gate voltage observed in a single-layer graphene sample transferred onto a fine-polished Na^{+}-SE substrate.
